# Comparison Performance of Visible-NIR and Near-Infrared Hyperspectral Imaging for Prediction of Nutritional Quality of Goji Berry (*Lycium barbarum* L.)

**DOI:** 10.3390/foods10071676

**Published:** 2021-07-20

**Authors:** Danial Fatchurrahman, Mojtaba Nosrati, Maria Luisa Amodio, Muhammad Mudassir Arif Chaudhry, Maria Lucia Valeria de Chiara, Leonarda Mastrandrea, Giancarlo Colelli

**Affiliations:** 1 Dipartimento di Scienze Agrarie, degli Alimenti e dell’Ambiente, Università di Foggia, Via Napoli 25, 71122 Foggia, Italy; danial.fatchurrahman@unifg.it (D.F.); mojtaba.nosrati@unifg.it (M.N.); mudassir.chaudhry@unifg.it (M.M.A.C.); maria.dechiara@unifg.it (M.L.V.d.C.); Leonarda.mastrandrea@unifg.it (L.M.); Giancarlo.colelli@unifg.it (G.C.); 2 Department of Agricultural Engineering, Faculty of Agricultural Technology, University of Brawijaya, Jl. Veteran, Malang 65145, Indonesia

**Keywords:** prediction, vitamin C, phenols, soluble solids, acidity

## Abstract

The potential of hyperspectral imaging for the prediction of the internal composition of goji berries was investigated. The prediction performances of models obtained in the Visible-Near Infrared (VIS-NIR) (400–1000 nm) and in the Near Infrared (NIR) (900–1700 nm) regions were compared. Analyzed constituents included Vitamin C, total antioxidant, phenols, anthocyanin, soluble solids content (SSC), and total acidity (TA). For vitamin C and AA, partial least square regression (PLSR) combined with different data pretreatments and wavelength selection resulted in a satisfactory prediction in the NIR region obtaining the R^2^_pred_ value of 0.91. As for phenols, SSC, and TA, a better performance was obtained in the VIS-NIR region yielding the R^2^_pred_ values of 0.62, 0.94, and 0.84, respectively. However, the prediction of total antioxidant and anthocyanin content did not give satisfactory results. Conclusively, hyperspectral imaging can be a useful tool for the prediction of the main constituents of the goji berry (*Lycium barbarum* L.).

## 1. Introduction

The goji berry (*Lycium barbarum* L.) is widely recognized for its outstanding health benefit, a fruit of the family of Solanaceae [1]. Originating from Asia, it was introduced in Europe in the 18th century for its famous benefits for health and medical properties [2]. Freshness is a quality attribute that determines the commercial values and sales of goji. A conventional method such as ultraviolet/visible spectrometry and HPLC can accurately determine the phytonutrients’ quality attributes such as antioxidant activity, total phenols, and multivitamins [3,4,5]). However, these analyses are time-consuming, and need expensive instruments and trained people, and as such cannot be used to assess the nutritional composition of individual fruit. Near-infrared spectroscopy has been utilized effectively to overcome these difficulties [6,7,8,9]). The hyperspectral imaging technique, which is a combination of the spectroscopic and imaging techniques, has been implemented due to its robustness of acquiring simultaneously the spectral and spatial information [6,7,8,9,10,11].

The hyperspectral imaging technique requires a data analysis approach which is an essential step in phytonutrient quality determination. Recently, spectral preprocessing, wavelength selection, and feature extraction, various modeling and model parameter optimization procedures, have been used to improve the accuracy of the determination [8,12,13]. Calibration models are crucial for the determination of phytonutrients. High accuracy and robust models are preferable because of their high potential for industrial application. Partial least square (PLS) combined with interval partial least square (iPLS) for wavelength selections has been proven to be a robust method for the prediction of chemical compositions using near-infrared spectroscopy on apples [14]. The application of the PLS regression model has also been successfully used for the prediction of six different maturity stages of tomatoes from green to red by using a portable visible and near-infrared spectrophotometer [14]. Hyperspectral imaging allowed users to discriminate the harvest time and to predict the internal content of soluble solids, phenols, and antioxidant activity of fennels [15], allowing them to create of a concentration map for each component. Furthermore, recently in dried black goji berry, Zhang et al. 2020 [11] successfully predicted the total anthocyanin, total flavonoid, and total phenols by using the hyperspectral image method combined with PLS and LS-SVM, and [16] successfully predicted antioxidant activity combined with a PLS regression model on dried black goji berry. However, available literature is lacking on research applications aimed to predict the nutritional content of fresh goji berry, and therefore the objective of this study was comparing the performance of the hyperspectral imaging method combined with PLSR in both region Vis-NIR and NIR to predict the concentration of SSC, TA, vitamin C (dehydroascorbic acid plus ascorbic acid), anthocyanins, total phenols, and total antioxidant activity.

## 2. Materials and Methods

### 2.1. Sample Preparation and Spectral Acquisition

**Figure 1:**
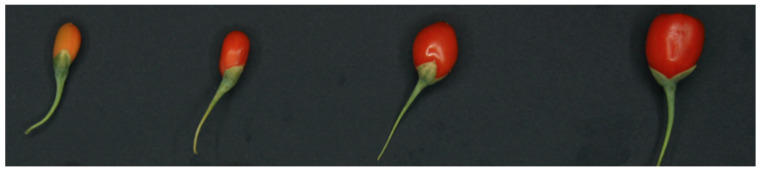
Four maturity stages of goji berry (Stage 1 to 4 from left to right).

The total amount of 3.6 kg of goji berry fruit (*Lycium barbarum* L.; Cultivar: sweet berry) grown in an open field in the Province of Castellaneta (Italy) was harvested conventionally by picking the fruit with its peduncle. Four maturity stages of the goji berry were harvested, starting from the early stage where fruit are still at the pinkish color with an average weight of 0.3 g, and average dimensions of approximately 9.89 mm and 7.23 mm for major and minor axis, respectively, to the mature stage where fruit are at a red color with an average weight of 1.3 g, possessing dimensions of 16.26 mm and 13.15 mm, respectively (Figure 1).

Damaged fruit were removed leaving 2.6 kg of sound fruit, after which fruit were scanned and classified based on the maturity stages, resulting in a total of 383 images (92 images for vitamin C, AA, and DHAA; 97 images for total phenol and total antioxidants; 97 images for anthocyanin; 97 images for SSC and TA); the images were then split into 2 data sets for the prediction model analysis (70% were used for the calibration data set and around 30% for the prediction data set). Approximately 2.5 g of the homogenized fruit sample from around 5 fruit were needed for individual chemical analysis; furthermore, as for the spectral analysis, a mean spectrum from those fruit was used.

### 2.2. Hyperspectral Image Acquisition

Hyperspectral image acquisition was done by using a hyperspectral line-scan scanner (Version 1.4, DV srl, Padova, Italy) consisting of two sensors, one in the visible near-infrared (Vis-NIR) region and the other in the near-infrared region (NIR). The region of VIS-NIR has a spatial resolution of 25,000 × 12,500 pixels/mm with a spectral resolution of 5 nm over a wavelength range of 400–1000 nm; however, in the NIR region, the spatial resolution was 7787.5 × 4000 pixels/mm and 5 nm spectral resolution covering the wavelength range of 900–1700 nm. In the case of Vis-NIR, a CCD camera was used, while a CMOS was used for NIR with 50 frames per second equipped with C-mount lenses. A cooled halogen lamp with a stabilized power source was used as the excitation system. The GigE vision was used as the interface with a 37° field of view (FOV). Image thresholding, masking, and the extraction of the average spectra were done under MATLAB with a self-developed code.

### 2.3. Chemical Analysis, and Partial Least Square Regression (PLSR)

#### 2.3.1. Determination of Vitamin C

Vitamin C, as the sum of Ascorbic acid (AA) and dehydroascorbic acid (DHAA) contents, was determined as described by [17], applying some modifications. Approximately 2.5 g of fruit tissue in 5 mL of methanol/water (5:95), plus citric acid (21 g L^−1^), EDTA (0.5 g L^−1^), NaF (0.168 g L^−1^), using an Ultraturrax (IKA, T18 Basic; Wilmington, NC, USA) were mixed for 1 min. The homogenate was filtered by using cheesecloth, while the pH was adjusted to 2.2–2.4 by the addition of 6 mol L^−1^ HCL. The analysis of HPLC was acquired after derivatization of DHA into the fluorophore 3-(1,2-dihydroxy ethyl) furol [3,4-b] quinoxaline-1-one (DFQ), with 1,2-phenylenediamine dihydrochloride (OPDA). Samples of 20 μL were analyzed with a HPLC (Agilent Technologies 1200 Series; Agilent, Waldbronn, Germany) equipped with a DAD detector and a binary pump, as described in [18]. AA, DHAA, and Vitamin C contents were expressed as g kg^−1^ of fresh weight.

#### 2.3.2. Determination of Anthocyanin

Total anthocyanin was determined by following a method introduced by [19]. Couples of discs (top cut) from fresh goji berries were taken (approx. 1 mm of thickness). The area was then calculated with an area of the ellipse formula A=a x b x π. Then, goji fruit discs were shaken in 3 mL of acidified methanolic solution (10 mL HCl/L) for 3 h at room temperature in the dark. Furthermore, the level of anthocyanin was determined based on the formula introduced by [20]:

$$\mathrm{Anthocyanin}={\mathrm{Absorption}}_{532\;\mathrm{nm}}-0.25\;\left({\mathrm{Absorption}}_{653\;\mathrm{nm}}\right)$$

The molar concentrations of anthocyanins/cm^2^ were acquired by dividing the optical density values by the molecular extinction coefficient of cyanidin (2.45 × 104), then divided by the area of the leaf discs. Hence, the results are expressed in mg of cyanidin per cm^2^ [1].

#### 2.3.3. Total Polyphenol and Antioxidant Activity

The determination of total phenol was done by using 2.5 g of goji berries homogenized in Ultraturrax (IKA, T18 Basic; Wilmington, NC, USA) for 1 min in 80% methanol: 20% water solution 2 mmol L^−1^ in sodium fluoride for 1 min. The resulted homogenate was then centrifuged under temperature 4 °C for 10 min at 9000 rpm. The method was done by following a protocol previously described by Cefola et al. (2010) [18]. The total phenols content was calculated based on the calibration curve of gallic acid per 100 g of fresh weight (mg GA 100 g^−1^). The determination of antioxidant was done by a method introduced by [21] with few modifications [18]. Fifty microliters of diluted samples were mixed with 0.950 mL of DPPH solution to initiate the reaction. The absorbance was measured at 515 nm after a 24 h incubation. Trolox was used as a standard, and the antioxidant activity was expressed in grams of Trolox equivalents per kg of fresh weight (TE g kg^−1^).

#### 2.3.4. Maturity Indexes

The determination of soluble solid content (SSC), and titrable acidity (TA), was done by using 5 berries placed in a falcon tube, then homogenized in an Ultra-Turrax (IKA T18 basic, Staufen, Germany), and filtered with two layers of cheesecloth (JC NONSTE SWAB 4040, China). The obtained juices were employed for direct reading of the SSC (%) using a digital refractometer (Atago N1, PR32-Palette, Tokyo, Japan), while 1 g juice samples were used for TA measurement by an automatic titrator (TitroMatic CRISON, Barcelona, Spain). The samples were titrated against a 0.1 mol L^−1^ NaOH solution up to a final pH of 8.1 and were expressed as a percentage of citric acid per 100 g sample.

#### 2.3.5. Partial Least Squares Regression (PLSR)

The PLS algorithm for the desired parameters prediction models was developed by using PLS toolbox (Eigenvector Research Inc. Wenatchee, WA, USA, version 7.2.5) working under MATLAB 2020b (version 9.9.0.1467703, MathWorks, Natick, MA, USA) as well as in HYPER-Tools (Version 3.0). HYPER-Tools works under the Matlab environment and can be freely downloaded at (https://www.hypertools.org/, accessed on 11 December 2020) [22]. The spectral data set was divided into calibration set and validation set based on the 70/30 ratio with 70% of the samples in the calibration data set and 30% of the samples reserved for external validation from the replicates of each acquisition interval. For the development of the PLSR calibration models, leave one out (LOO) cross-validation was applied. The optimum numbers of latent variables were chosen by using a convenient technique described by Haaland and Thomas, 1988 [23]. It consists of computing the ratios between the PRESS (Predicted Residual Error Sum of Squares) values and the minimum one. These PRESS ratios play the role of variance ratios (analogous to the statistical F parameter) so that they can be associated with a probability *p*. The proposal, based on empirical results, is that the number of latent variables to be selected for which the associated probability *p* is more than 0.75 [24]. The accuracy of the calibration models was accessed by visualizing the root mean square error for calibration (RMSEC) and cross-validation (RMSECV). As the first approach, different pre-treatments’ techniques by using all the wavelengths were attempted, including smoothing, mean centering, 1st and 2nd derivatization, and their combinations. Then, after the development of these models, the most significant variables were selected based on modified interval-PLS [24]. In the modified interval-PLS method, the full spectral range was divided into sub-regions of specific variables, and then in each of these intervals, a separate model was formulated and evaluated by removing variables belong to the intervals. Finally, the eliminated intervals driving to improve accuracy were discarded from the full ranges.

Moreover, to detect the presence of outlying samples—those whose nominal analyte concentration significantly deviates from the prediction when they are left out from the set—the following indicator, estimating the summation of deviations over the cross-validation process, was used [23]:

(1) $$F_{y}\left(i\right)=\frac{\left(I-1\right){\left(y_{pred,\;i}-y_{nom,\;i}\right)}^{2}}{\sum_{i\neq j}{\left(y_{pred,\;j}-y_{nom,\;j}\right)}^{2}}$$

where *y_pred,i,_* and *y_nom,i_* are the predicted and nominal value for the left-out sample during cross-validation; *y_pred,j,_* and *y_nom,j_* are the corresponding values for the remaining samples; and *I* is the number of samples. The degrees of freedom for studying the significance of *F_y_(i)* are 1 and (*I* − 1) for the numerator and denominator, respectively. Self-developed MATLAB code was used for modified interval-PLS and sampling to develop the PLSR approach.

All models were finally tested on the external data set to assess prediction performance. Moreover, for the best prediction models, the R^2^ of calibration, cross validation, prediction, and root mean square error of prediction (RMSEP) were also assessed.

#### 2.3.6. Mapping of Internal Constituents

Mapping of the internal constituent on the different stages of the goji berry was done by firstly extracting an average spectrum from the pixels of the fruit image sample by considering that the mean spectrum corresponds to the average of a constituent of the fruit. Thus, based on the PLSR models that were developed from the calibration data set, the level of an internal constituent of the goji berry was predicted in order to show the distribution of internal constituents of each goji fruit.

## 3. Results and Discussion

### 3.1. Spectral and Spatial Profile

**Figure 2:**
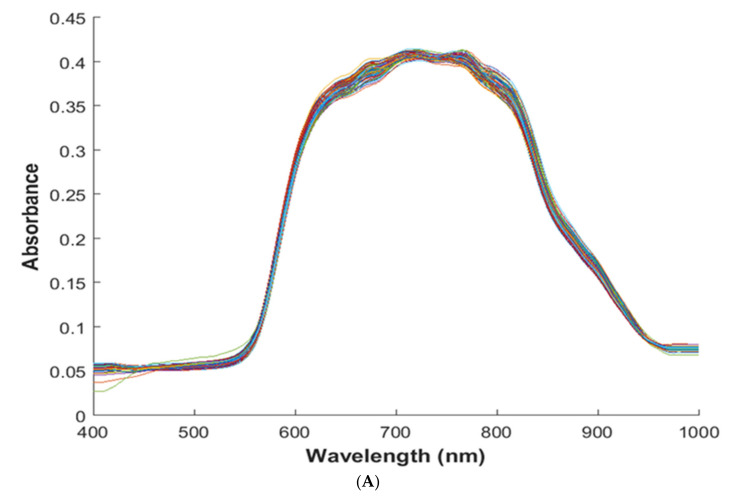

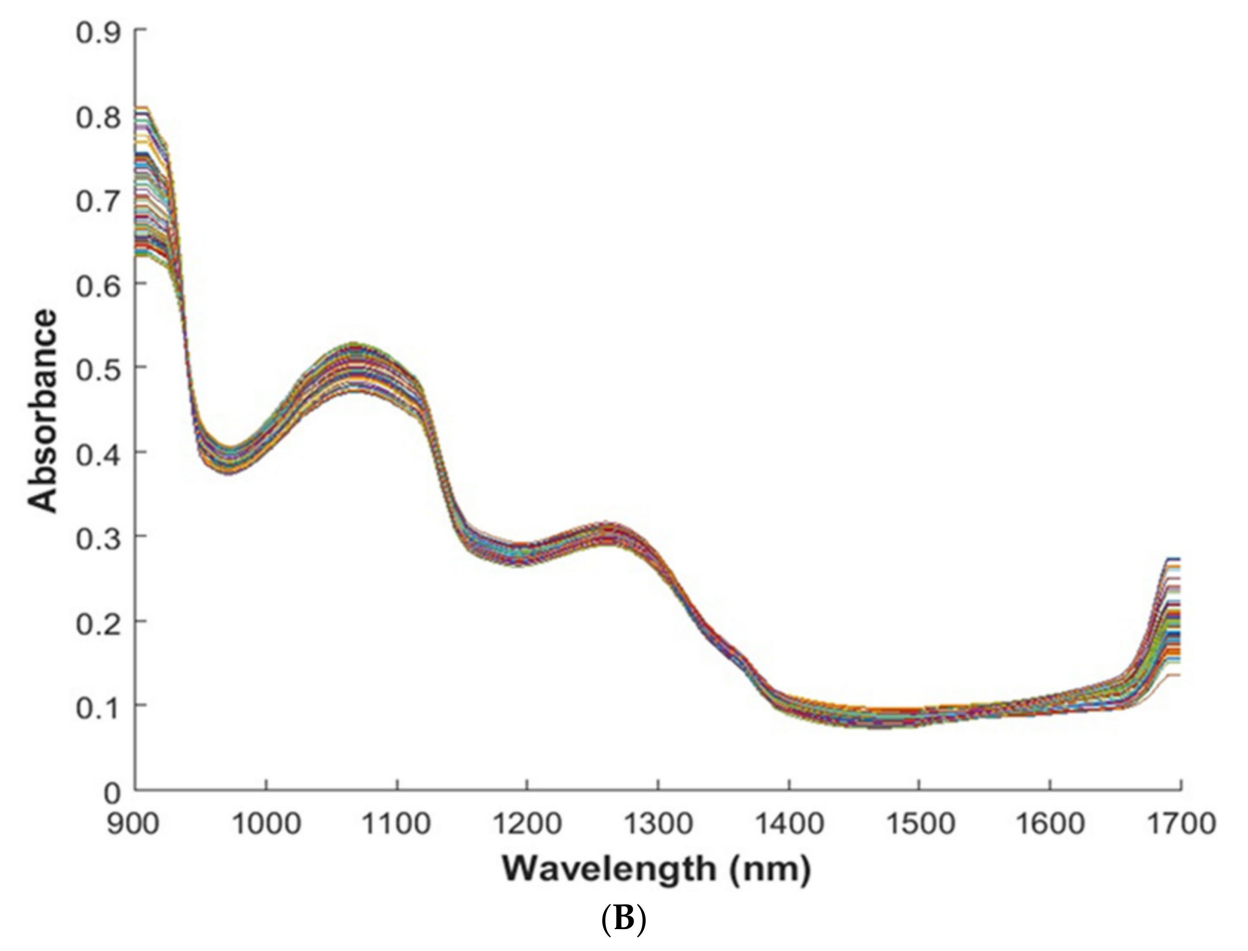
Pre-processed absorbance spectra of goji berry at the wavelength range of (**A**) VIS-NIR 400–1000 nm and (**B**) NIR 900–1700 nm.

Figure 2 shows the preprocessed reflectance spectra of the goji berry in both regions of the Visible Near-Infrared and Near-Infrared regions. However, the spectra obtained cannot be directly used for the determination of specific chemical constituents, since each spectrum reflects the complex constituent information. Data analysis approaches were performed to explore the relationship between spectra and vitamin C, AA, DHAA, total phenols, anthocyanin, SSC, and TA of the goji berry.

Figure 2 depicts the VIS-NIR and NIR spectra profile of the goji berry. In the VIS-NIR, peaks correlated to the colors which are associated with phenols, carotenoids, anthocyanin, and chlorophyll compounds [25,26,27]. Additionally, some peaks found in the region of VIS-NIR at 900–970 nm are reported to be associated with overlapped peaks of starch, cellulose, sucrose, and water, and in particular, peaks from 900 nm to 920 nm are reported to correlate with starch and cellulose [28,29]. Regarding the NIR region, peaks generally represent the constituents of water and vitamin C. It has been reported that the peaks with ranges of 900–1000 nm and 1400–1500 nm are peaks corresponding to the constituent of water [30,31]. Furthermore, peaks at 850, 1000, 1210, 1360, 1460, 1580, 1650 nm have been reported to correlate with vitamin C in powdered mixtures and solutions [31], as well as in spectra acquired with HIS on whole rocket leaves [32].

### 3.2. Comparison of Prediction Model between Spectra Range VIS-NIR and NIR

In Table 1, the mean values and respective range of composition for each nutritional quality parameter analyzed in this study are shown. Thanks to the different maturity stages, a large variation in the minimum and the maximum values for the chemical parameters were obtained to enlarge the interval of variation of the calibration models. The results obtained in this study are in a line with previous reports on the internal constituents of the goji berry from different maturity stages that as the vitamin C level increased, the fruits are more ripe and become softer, followed by the increase in SSC and TA [33,34]. Regarding total phenol, antioxidant, and anthocyanin levels of the goji berry from different maturity stages, to the best of our knowledge there was not any report available yet; however, our results on the ripe stage are in accordance with the previous reports [1,3].

In this study, the potentiality for both wavelengths ranges in VIS-NIR and NIR for the prediction model of vitamin C, ascorbic acid (AA), dehydroascorbic acid (DHAA), total antioxidant activity, total phenols, anthocyanin, soluble solid content (SSC), and total acidity (TA) are compared. In general VIS-NIR and NIR spectra regions, both are giving reliable predictions of vitamin C, AA, total phenols, SSC, and TA, while the prediction for DHAA is not satisfying (Table 2 and Table 3). However, comparing both regions VIS-NIR and NIR for the prediction of the nutritional value of the goji berry, the best performance of the prediction for total phenols, SSC, and TA are best given in the spectra region of VIS-NIR, whereas the best prediction performance for vitamin C and AA is best given in the spectra region of NIR. It is suggested that the prediction of total phenols is best explained in the visible region since the peaks containing the compounds are in the VIS-NIR region at the range of 400–500 nm [26]. Particularly, it is reported that ferulic acid which is the dominant phenolic of the goji berry [35] shows a maximum reflectance peak at 450 nm [36]. Regarding the SSC, the peaks containing starch, cellulose, and sucrose are also in the VIS-NIR region of 890–920 nm [28,29]. As for TA, slightly better results were obtained in the VIS-NIR region, indicating that the spectra that contribute to detecting acids are covering both regions of VIS-NIR and NIR. A previous study reported that citric acid absorbance is represented in the wide region between 900 nm and 1650 nm [37], while in this study we concluded that most of the information is contained within 900–1000 nm. Another explanation for this result is that in addition to spectral information directly related to acid content, in the VIS-NIR range other wavelengths are indirectly contributing to detect the acidity, being related to the maturity of the fruit, resulting in better performance for this region, compared to NIR. Moreover, for vitamin C and AA, both ranges were giving satisfying results, but in this case, NIR results showed a slightly better performance compared to the VIS-NIR region at 400–1000 nm. This can confirm what was already found for rocket leaves, showing that major peaks correlating with vitamin C are in the NIR region (1000, 1210, 1360, 1460, 1580, 1650 nm), while only one peak (850 nm) is found in the VIS-NIR region [32,33].

The PLSR models yielded reliable and satisfying results for the Vitamin C and AA, phenols, SSC, and TA. Different pre-treatments techniques were applied including smoothing, mean centering, 1st and 2nd derivatization, and their combinations (Table 2 and Table 3). Besides, to improve the performance after the development of these models, the most significant variables were selected based on modified i-PLS for each parameter and tested to an external data set. Thus, the best prediction results are explained in terms of regression R^2^, the root mean square error for calibration (RMSEC), leave one out cross-validation (RMSECV), and root mean square error for prediction (RMSEP) in NIR and Vis-NIR range, respectively (Table 4). Particularly when R^2^ and errors were giving different indications, models with the lowest prediction error were selected.

**Figure 3:**
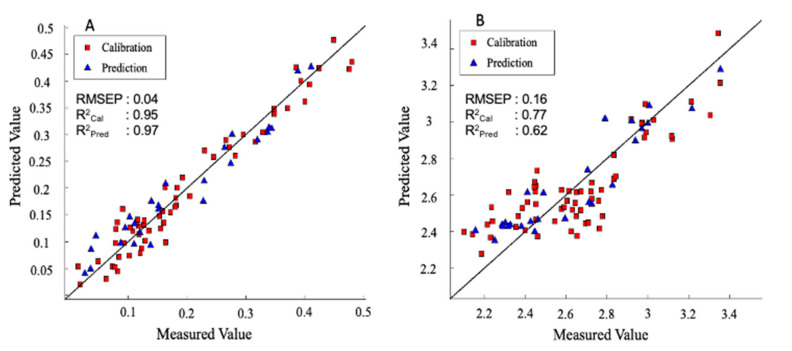
(**A**) AA model (NIR range) and (**B**) Total phenols model (VIS-NIR range): PLS regression plot for predicted vs. measured values.

As can be seen in Table 2, Table 3 and Table 4, the best model performance was chosen based on the selection of both the lowest RMSEP [10] and the highest R^2^. In the case of the Ascorbic Acid (AA), encouraging results over 95 samples were obtained with 12 optimal LV in the NIR range, after pre-treating the data using a combination of smoothing, logarithm, first derivative, and mean centering. This pre-processing allowed obtaining the lowest error in cross validation (0.54 g kg^−1^ with an R^2^ of 0.65), even if a model with a much higher R^2^ could be selected (0.94 but with an error of 0.55 g kg^−1^). Selecting the most effective 84 wavelengths and removing outlier samples, the improved model resulted in 11 LV and enhanced performance in calibration R^2^_Cal_ = 0.97, and with R^2^_pred_ = 0.91 and RMSEP of 0.04 g kg^−1^, as shown in Figure 3A. The most effective wavelengths for the prediction of AA are given in Table 4. Compared to a study on the prediction of AA in bell pepper, which is the same family as goji berry, we obtained a better performance where the prediction of AA yielded performance only with R^2^_pred_ = 0.70 with an error of 0.18 g kg^−1^ [38]. Furthermore, in the case of DHAA, we did not get a satisfactory result (Table 3), and as a consequence, a little worse performance of the prediction model for vitamin C (sum of AA and DHAA) was reached.

In particular, for vitamin C the best calibration model performance was obtained in the NIR range, with a data set comprised of 95 samples, and applying a combination of smoothing, logarithm, first derivative followed by mean centering as pre-treatment (Table 3). After selecting the most effective wavelength and removing outlier samples, optimal LVs were reduced to 12, enhancing the performance in calibration and prediction, yielding to R^2^_Cal_ = 0.96 and R^2^_pred_ = 0.91 with RMSEP of 0.04 g kg^−1^ (Table 4). Furthermore, the most effective wavelengths used for the prediction of vitamin C belonged to the following intervals, 1475–1495 nm, 1525–1545 nm, and 1600–1650 nm. As to the best of our knowledge, there is not any available report yet for the prediction of vitamin C in goji berries, but comparing to the prediction of vitamin C with other fruit from previous papers, this model gained better accuracy. As in an intact tomato, in the same family of Solanaceae, [39] reported the prediction result of R^2^_pred_ = 0.82 and RMSEP of 0.17 g kg^−1^ by using the whole wavelength range of 930 nm to 1650 nm, while for the chili pepper obtained R^2^_cal_, R^2^_pred_, and RMSEP values of 0.95, 0.8, and 0.01 g kg^−1^ [40]. Moreover, for other fruit, lower performances are reported; for the apple, an R^2^_pred_ of 0.81 and RMSEP of 0.05 g kg^−1^ [41], and for the orange, an R^2^_cal_ = 0.82 and R^2^_pred_ = 0.72 with RMSEP 0.9 g kg^−1^ [42], have been reported, respectively.

Regarding total phenols in the goji berry, the best model acquired was in the VIS-NIR region; the data set comprised 95 samples, and the best calibration model was obtained by applying a combination of smoothing, logarithm, second derivative, and mean centering as pre-treatment. Furthermore, selecting the most effective wavelength and removing outlier samples resulted in reducing the optimal LV to 2 and enhancing the performance in calibration and prediction, yielding an R^2^_Cal_ = 0.77 and R^2^_pred_ = 0.62 with RMSEP of 0.16 g kg^−1^ respectively. The PLS regression plot is given in Figure 3B. Furthermore, the most effective wavelength range was shown to be in the wavelength intervals of 425–520 nm and 725–995 nm. This result explained that the major information for the most abundant phenolic compounds in the goji berry is ferulic acid which has the absorbance in the visible region [36,37]. However, a different result was reported in dried black goji berry (*Lycium ruthenicum* Murr.) where the prediction was best yielded in the NIR region with the R^2^_Cal_, R^2^_pred_, and RMSEP of 0.83, 0.84, 3.07 g kg^−1^, respectively [10]. As for black goji berry, the most abundant phenolic compounds are known to be coumaric and caffeic acids [43,44]. The most important peaks in the NIR region fall between 1100 nm and 1170 nm and between 1410 nm and 1480 nm [45]. Besides, it should be noted that Zhang et al. (2020) used dried goji berry used by [10], leading to a high concentration of phenolics. As we can observe, despite the higher R^2^ reported by these authors, the RMSEP was higher than in the present study. The error of prediction is, in fact, more important than R^2^ and should be compared to the standard error of laboratory (SEL). As for phenolics, RMSEP was in line with the value calculated for the laboratory destructive measure of total phenols which, in our case, is 0.1 g kg^−1^. It is reported that the value of RMSEP should be considered excellent if it is not higher than 1.5 times the SEL and good if around 2–3 times of laboratory error [45], thus confirming the robustness of the prediction model.

**Figure 4:**
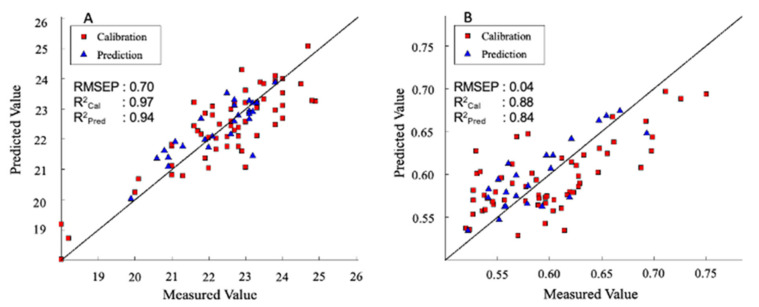
(**A**) SSC (VIS-NIR range) and (**B**) TA (VIS-NIR range): PLS regression plot for predicted vs. measured values.

**Figure 5:**
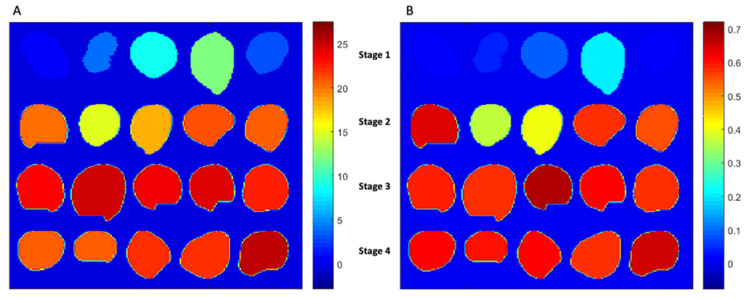
Prediction map of SSC (VIS-NIR range) (**A**) and TA (VIS-NIR range) (**B**).

As for SSC, the prediction model comprises 97 samples, elaborated in the VIS-NIR range, combining the smoothing, second derivative, followed by mean centering pre-treatments. Selecting the most effective wavelengths and removing outlier samples resulted in enhancing the performance in calibration and prediction yielding R^2^_Cal_ = 0.97 and R^2^_pred_ = 0.94 with RMSEP of 0.70. The PLS regression plot is shown in Figure 4A, whereas the most effective wavelengths for the prediction model are shown in Table 4. These results are slightly better than a similar study on the prediction of total sugar content in the goji berry; however, in this research, the prediction model was done by using FT-NIR spectroscopy, and the total sugar was measured by the quantification of glucose, reaching performance with R^2^_Cal_ = 0.97 and R^2^_pred_ = 0.92 with RMSEP of 0.9 [46]. Figure 5A depicts the prediction of the concentration of SSC from fruit over different maturity stages. It can be seen that goji berry fruit has a lower SSC concentration at approximately 4% in stage 1 and increasing to the level of concentration of 25% in stage 4, respectively. Regarding TA, the best PLSR model was developed using the VIS-NIR range, combining smoothing, second derivative, and mean centering. Selecting the most effective wavelengths and removing outlier samples resulted in reducing the optimal LV to 3 and enhancing the performance in calibration and prediction (R^2^_Cal_ = 0.89 and R^2^_pred_ = 0.84 and RMSEP of 0.04%). The PLS regression plot for TA is shown in Figure 4B. This is the first attempt of TA prediction in the goji berry with spectral data. Comparing these results with the prediction of TA in the grape tomato, despite the lowest R^2^_Cal_ and R^2^_pred_, we had the smallest prediction error (RMSP 0.04% vs. 0.072%) [47]. Furthermore, Figure 5B expresses the concentration of TA in the goji berry across four different maturity stages. Finally, it needs to be taken into some consideration that the laboratory error of SSC and TA was 0.392 and 0.02, respectively, which is confirming the robustness of the models. Finally, as for total antioxidant activity, and total anthocyanin, results were very poor. As for anthocyanins, it is possible that the signal due to the content of these phytonutrients is very low compared to the water and main constituents, since their content is lower even if compared to phenolics and vitamin C. Finally, antioxidant activity is correlated with the content of different compounds, but its measurement is the results of the addition of a free radical being reduced in an oxidizing medium [48], so it is completely understandable that no direct correlation with reflectance spectra exists since the response is chemically activated in the reference essay.

## 4. Conclusions

The potential of hyperspectral imaging in both VIS-NIR and NIR regions, together with multivariate data analysis, was evaluated for non-destructive determination of the internal composition of the intact goji berry. In general, the result from the prediction model in the VIS-NIR region can be used to predict total phenols, SSC, TA, showing better performance than in the NIR region. Despite the unsuitable prediction of DHAA, anthocyanin, and total antioxidant, the results obtained in the current study are very promising for the fast-quality evaluation of goji berry fruit, since vitamin C, total phenols, SSC, and TA are the most relevant parameters related to fresh goji berry composition and consumer acceptance.

## Figures and Tables

**Table 1:** Range values and statistical distribution of internal constituents’ quality attributes of fresh goji berries.

Parameters	Min	Max	Mean	Standard Deviation
Vitamin C (Vitamin C g kg^−1^)	0.13	0.65	0.31	0.14
Ascorbic Acid (AA) (Ascorbic Acid g kg^−1^)	0.02	0.48	0.20	0.12
Dehydroascorbic Acid (DHAA) (DHAA g kg^−1^)	0.04	0.18	0.11	0.04
Antioxidant Activity (Trolox equivalent g kg^−1^)	1.63	3.29	2.31	0.41
Total Phenols (gallic-acid g kg^−1^)	2.09	3.37	2.62	0.32
Anthocyanin (Cyanidin mg cm^−2^)	0.67	1.35	1.05	0.17
Soluble Solid Content (SSC) (%)	6.50	25.90	21.62	3.48
Total Acidity (TA) (%)	0.11	0.92	0.56	0.15

**Table 2:** Calibration statistics for the PLSR models of the internal constituents of fresh goji berries (SM = Smoothing, Dev = derivative, MC = mean centering) in the VIS-NIR region (400–1000 nm).

Parameter	Pre-Treatment	No. Var	No. Sample	LVs	R^2^_Cal_	RMSEC	R^2^_CV_	RMSECV
Vitamin C	SM + MC	121	92	10	0.79	0.05	0.69	0.07
	SM + 1st Dev + MC	121	92	5	0.60	0.06	0.56	0.07
	SM + 2nd Dev + MC	121	92	5	0.84	0.05	0.76	0.07
AA	SM + MC	121	92	13	0.89	0.03	0.64	0.05
	SM + 1st Dev + MC	121	92	7	0.75	0.04	0.64	0.05
	SM + 2nd Dev + MC	121	92	5	0.75	0.04	0.69	0.05
DHAA	SM + MC	121	92	3	0.31	0.03	0.31	0.03
	SM + 1st Dev + MC	121	92	1	0.36	0.03	0.40	0.03
	SM + 2nd Dev + MC	121	92	1	0.39	0.03	0.42	0.03
Total Antioxidant	SM + MC	121	97	4	0.21	0.32	0.22	0.35
	SM + 1st Dev + MC	121	97	3	0.31	0.30	0.31	0.34
	SM + Log + 1st Dev + MC	121	97	3	0.39	0.25	0.37	0.29
Phenols	SM + MC	121	97	2	0.37	0.26	0.40	0.27
	SM + 1st Dev + MC	121	97	5	0.36	0.19	0.33	0.23
	SM + Log + 2nd Dev + MC	121	97	2	0.41	0.23	0.44	0.24
Anthocyanin	SM + 1st Dev + MC	121	97	4	0.14	0.15	0.15	0.16
	SM + 2nd Dev + MC	121	97	2	0.18	0.16	0.18	0.17
	SM + Log + 2nd Dev + MC	121	97	1	0.15	0.16	0.16	0.16
SSC	SM + 1st Dev + MC	121	97	6	0.82	0.92	0.73	1.15
	SM + 2nd Dev + MC	121	97	4	0.79	0.98	0.69	1.25
	SM + Log + 1st Dev + MC	121	97	6	0.85	0.96	0.75	1.2
TA	SM + 2nd Dev + MC	121	97	4	0.43	0.06	0.43	0.09
	SM + Log + 1st Dev + MC	121	97	5	0.52	0.06	0.48	0.07
	SM + Log + 2nd Dev + MC	121	97	4	0.55	0.06	0.51	0.07

**Table 3:** Calibration statistics for the PLSR models of the internal constituents of fresh goji berries (SM = Smoothing, Dev = derivative, MC = mean centering) in the NIR region (900–1700 nm).

Parameter	Pre-Treatment	No. Var	No. Sample	LVs	R^2^_Cal_	RMSEC	R^2^_CV_	RMSECV
Vitamin C	SM + 1st Dev + MC	161	95	11	0.70	0.05	0.67	0.06
	SM + Log + 1st Dev + MC	161	95	17	0.65	0.03	0.64	0.06
	SM + Log + 2nd Dev + MC	161	95	10	0.78	0.04	0.70	0.06
AA	SM + 1st Dev + MC	161	95	13	0.94	0.04	0.81	0.06
	SM + Log + 1st Dev + MC	161	95	12	0.65	0.03	0.54	0.05
	SM + Log + 2nd Dev + MC	161	95	10	0.65	0.04	0.57	0.06
DHAA	SM + MC	161	95	4	0.22	0.03	0.26	0.03
	SM + 2nd Dev + MC	161	95	3	0.20	0.03	0.25	0.03
	SM + Log + 1st Dev + MC	161	95	3	0.21	0.03	0.26	0.03
Total Antioxidant	SM + MC	161	97	11	0.21	0.27	0.22	0.34
	SM + Log + 1st Dev + MC	161	97	3	0.22	0.32	0.27	0.35
	SM + Log + 2nd Dev + MC	161	97	2	0.27	0.33	0.34	0.34
Phenols	SM + 1st Dev + MC	161	97	8	0.39	0.18	0.43	0.22
	SM + Log + 1st Dev + MC	161	97	3	0.41	0.22	0.50	0.24
	SM + Log + 2nd Dev + MC	161	97	4	0.34	0.21	0.42	0.22
Anthocyanin	SM + MC	161	97	1	0.04	0.16	0.05	0.17
	SM + Log + 1st Dev + MC	161	97	1	0.05	0.16	0.06	0.17
	SM + Log + 2nd Dev + MC	161	97	1	0.06	0.16	0.07	0.17
SSC	SM + MC	161	97	10	0.14	1.32	0.16	1.57
	SM + 1st Dev + MC	161	97	7	0.19	1.38	0.22	1.59
	SM + Log + 2nd Dev + MC	161	97	8	0.15	1.38	0.16	1.76
TA	SM + 1st Dev + MC	161	97	3	0.42	0.07	0.54	0.08
	SM + 2nd Dev + MC	161	97	4	0.40	0.07	0.52	0.08
	SM + Log + 1st Dev + MC	161	97	7	0.34	0.07	0.40	0.08

**Table 4:** Prediction statistics for the best PLSR models of the internal constituents of fresh goji berries in the VIS-NIR region (400–1000 nm) and NIR region (900–1700 nm).

Parameter	Effective WaveLength Range (nm)	No. Sample	No. Variables	LVs	R^2^_Cal_	RMSEC	R^2^_CV_	RMSECV	R^2^_pred_	RMSEP
Vitamin C	1475–1495 1525–1545 1600–1650	85	18	12	0.86	0.03	0.84	0.04	0.96	0.04
AA	925–945 975–1120 1175–1270 1300–1320 1425–1445 1475–1495 1525–1545 1600–1620	85	72	11	0.95	0.02	0.91	0.04	0.97	0.04
Total Phenols	425–520 725–995	87	73	2	0.77	0.16	0.61	0.17	0.62	0.16
SSC	400–495 525–545 625–670 850–895 925–970	89	50	4	0.97	0.75	0.95	0.91	0.94	0.70
TA	400–445 600–620 775–795 825–895	87	31	3	0.88	0.04	0.87	0.04	0.84	0.04

## Data Availability

The data presented in this study are available on request from the corresponding author.
